# Interactions and Feedbacks in E-Cadherin Transcriptional Regulation

**DOI:** 10.3389/fcell.2021.701175

**Published:** 2021-06-28

**Authors:** Miguel Ramirez Moreno, Przemyslaw A. Stempor, Natalia A. Bulgakova

**Affiliations:** ^1^Department of Biomedical Science and Bateson Centre, The University of Sheffield, Sheffield, England; ^2^SmartImmune Ltd., Cambridge, United Kingdom

**Keywords:** adhesion, JAK/STAT, heterochromatin, HP1, PAR-3

## Abstract

Epithelial tissues rely on the adhesion between participating cells to retain their integrity. The transmembrane protein E-cadherin is the major protein that mediates homophilic adhesion between neighbouring cells and is, therefore, one of the critical components for epithelial integrity. E-cadherin downregulation has been described extensively as a prerequisite for epithelial-to-mesenchymal transition and is a hallmark in many types of cancer. Due to this clinical importance, research has been mostly focused on understanding the mechanisms leading to transcriptional repression of this adhesion molecule. However, in recent years it has become apparent that re-expression of E-cadherin is a major step in the progression of many cancers during metastasis. Here, we review the currently known molecular mechanisms of E-cadherin transcriptional activation and inhibition and highlight complex interactions between individual mechanisms. We then propose an additional mechanism, whereby the competition between adhesion complexes and heterochromatin protein-1 for binding to STAT92E fine-tunes the levels of E-cadherin expression in *Drosophila* but also regulates other genes promoting epithelial robustness. We base our hypothesis on both existing literature and our experimental evidence and suggest that such feedback between the cell surface and the nucleus presents a powerful paradigm for epithelial resilience.

## Introduction

Cells in epithelia adhere to both a common substrate and neighbouring cells (Hagios et al., 1998). The molecules mediating this adhesion (cell adhesion molecules, CAMs) are the driving force behind the tissue architecture (Gumbiner, 1996; Buckley et al., 1998; Hagios et al., 1998; Schock and Perrimon, 2002), whereas adhesion defects often occur during tumour formation and metastasis (Zetter, 1993; Janiszewska et al., 2020). Among CAMs, the calcium-dependent adhesion (cadherin) proteins mediate the direct cell–cell adhesion through homophilic binding of extracellular domains (Perez and Nelson, 2004; Nishiguchi et al., 2016). In this perspective, we focus on Epithelial cadherin (E-cadherin, E-cad), as in epithelial cells, it plays a major role in tissue formation and maintenance (Takeichi, 1977; Halbleib and Nelson, 2006; Nelson, 2008; Kaszak et al., 2020). In contrast, other cadherins such as P-cadherin do also contribute to cell–cell adhesion in epithelia but their actions are restricted to specific areas and developmental stages (Paredes et al., 2012).

The strength of adhesion correlates with the number of E-cad molecules on cell surfaces (Chu et al., 2004). Consequently, the regulation of E-cad surface levels in epithelia is instrumental in many processes, and even mild changes in E-cad levels profoundly affect many processes such as cell rearrangements, proliferation, and tissue architecture (Ciesiolka et al., 2004; Lecuit and Yap, 2015; Mohan et al., 2018; Greig and Bulgakova, 2020). Multiple mechanisms regulate E-cad surface levels including intracellular trafficking, transcriptional regulation, post-translational modifications, and protein degradation (Palacios et al., 2005; Bertocchi et al., 2012; Serrano-Gomez et al., 2016; Brüser and Bogdan, 2017; Cai et al., 2018). In this perspective, we discuss transcriptional regulation, as the decrease in E-cad protein levels often correlates with reduced mRNA abundance (Bringuier et al., 1999).

## Transcriptional Repression

**FIGURE 1:**
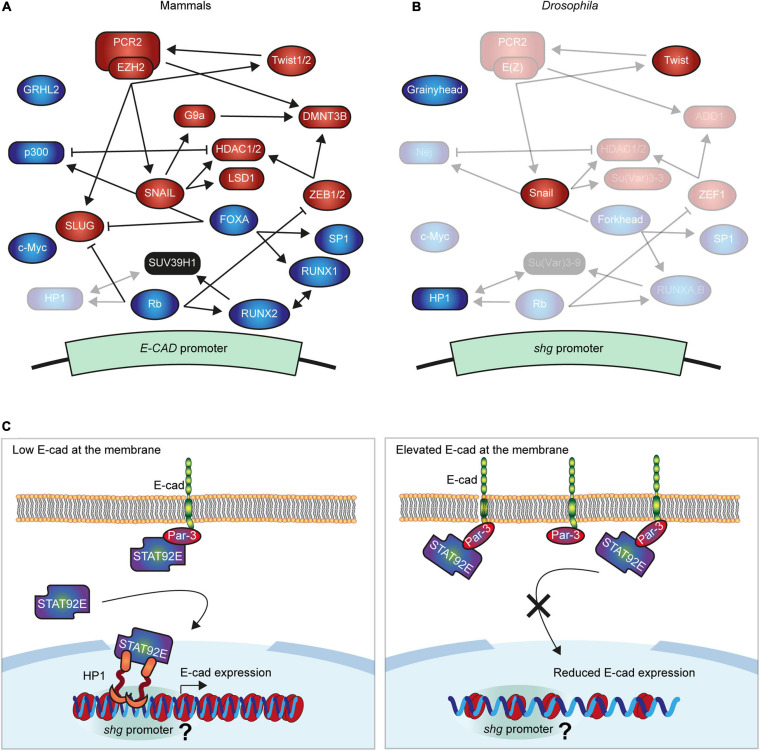
Molecular mechanisms regulating E-cadherin gene expression. **(A,B)** Summary of the best known negative and positive regulators of E-cad in mammals **(A)** and *Drosophila* **(B)**. The expression of E-cad gene (*shotgun*, *shg*, in *Drosophila*) is controlled by transcription factors (ellipses), which inhibit (red) or promote it (green) and interact with the E-cad promoter with varying affinities. Several further proteins modify chromatin at the promoter (squircles) silencing the E-cad expression (red) or increasing it (green). These proteins cooperate, compete, and regulate each other, establishing the intricated network that fine-tune the expression of E-cad. In panel **(B)** the non-conserved proteins were removed while conserved maintained their relative positions but their names were replaced with those of the *Drosophila* orthologs. The regulatory roles of transparentized proteins in panels **(A,B)** were not demonstrated in the respective systems. **(C)** The proposed model of E-cad modulation of its expression. A subpool of STAT92E translocates into the nucleus independently of the canonical JAK/STAT signalling, where it interacts with heterochromatin protein 1 (HP1). Within euchromatin, HP1 localises at specific loci corresponding to gene promoters, including that of the *shg* gene. Concurrently, STAT92E is recruited to the cell surface by E-cad and its binding partner Par-3. Therefore, elevated levels of E-cad outcompete STAT92E from the nucleus inhibiting its function in heterochromatin formation and promoter regulation. As the result, elevated E-cad at the cell surface reduces *shg* expression, ultimately restoring its levels. It is unclear whether this is accompanied by a change in chromatin organisation around *shg* promoter, indicated by ‘?.’

The best-studied regulation of E-cad transcription in mammalian cells is silencing by transcription factors (TFs) including SNAIL, SLUG (also known as SNAI1 and SNAI2 in mammals), ZEB1/2, and Twist1/2 (Figure 1A), all of which directly bind conserved E-boxes (CANNTG sequences) in the E-cad promoter (Giroldi et al., 1997; Comijn et al., 2001; Bolós et al., 2003; Wong et al., 2014; Vergara et al., 2016; Russell and Pranjol, 2018). All events of epithelial-to-mesenchymal transition (EMT) during development involve at least one of these TFs, and they were all linked to the tumour progression (Gasparotto et al., 2011; Muenst et al., 2013; da Silva et al., 2014; Vergara et al., 2016; Yang et al., 2020). Despite the overall similarity in the action of these TFs, their different affinities and variable expression allow for a dynamic but tightly regulated expression of E-cad (Bolós et al., 2003; Eger et al., 2005; Schmidt et al., 2005; Vesuna et al., 2008; Mazda et al., 2011). Regulation of E-cad transcription by these TFs is extremely conserved across evolution. While *Drosophila* and mammalian E-cad differ in their extracellular domains and are products of independent evolution from a common N-cadherin-like ancestor, transcription of the *Drosophila* E-cad (encoded by the *shotgun* gene) is also inhibited by the Snail and Twist TFs (Figure 1B; Oda et al., 1998, 2005; Oda and Takeichi, 2011; Nishiguchi et al., 2016). This could be either due to this regulation already being present in the ancestor or a result of the parallel evolution due to the same functional requirements. In *Drosophila*, while Snail represses E-cad during gastrulation in the embryo, it does not inhibit it in the adult midguts (Oda et al., 1998; Campbell et al., 2019), potentially due to the combinatorial action of other regulators (Figure 1B).

Multiple epigenetic mechanisms also inhibit E-cad expression in mammalian cells. Hypermethylation of the large CpG island around the promoter is frequently linked with loss of E-cad expression (Yoshiura et al., 1995; van Roy and Berx, 2008). This DNA hypermethylation is often accompanied by histone modifications associated with inactive chromatin (Serrano-Gomez et al., 2016). Most notably, polycomb repressive complex 2 (PRC2) is recruited to the E-cad promoter where its key component, enhancer of zeste homolog 2 (EZH2), places the repressive H3K27me3 mark, deactivating E-cad transcription (Viré et al., 2006; Cao et al., 2008; Fujii and Ochiai, 2008). The histone methyltransferase G9a places the repressive H3K9me2/3 marks on the E-cad promoter (Dong et al., 2012). Histones are subjected to other post-translational modifications that contribute to E-cad silencing, such as the removal of the H3K4me2 mark by the lysine-specific demethylase 1 (LSD1) (Lin et al., 2010), or H3 and H4 deacetylation by the histone deacetylases 1 and 2 (HDAC1/2) (Peinado et al., 2004a). Histone phosphorylation and ubiquitination were also suggested to alter transcription of the mammalian E-cad (Surapaneni et al., 2020; Wang et al., 2020). While there is only a low level of DNA methylation in *Drosophila* (Lyko et al., 2000) other epigenetic mechanisms are conserved (Figure 1B) and might, similarly to Snail and Twist, regulate E-cad transcription in invertebrates.

These mechanisms of E-cad silencing closely interact with each other (Figure 1A). For example, SNAIL recruits the repressive complex containing LSD1 and HDAC1/2 (Peinado et al., 2004a; Lin et al., 2014), which may facilitate the subsequent recruitment of PRC2 (Ai et al., 2017; Jin et al., 2017, p. 1). SNAIL also recruits G9a to the E-cad promoter, whereas both G9a and PRC2 interact with the DNA methyltransferases DNMT1/3A/3B, and thus can promote the CpG methylation (Viré et al., 2006; Dong et al., 2012; Lin et al., 2014). Twist1 can also promote the recruitment of PRC2 (Cakouros et al., 2012; Malouf et al., 2013), whereas ZEB1 recruits histone deacetylases, HDAC1/2, and possibly DNMT3B (Aghdassi et al., 2012; Zhang et al., 2019). Concurrently, EZH2 facilitates the expression of SNAIL and SLUG through an unknown mechanism (Zhang et al., 2017), and increases Twist expression by suppressing the miR-361 (Ihira et al., 2017). Through these interactions, we suggest that individual mechanisms act together to ensure the robust silencing of the E-cad.

## Transcriptional Activation

A fluctuation in the activity of a repressive mechanism, for example, due to random changes in fundamentally stochastic expression of regulatory genes, could lead to the E-cad loss (Raj and van Oudenaarden, 2008; Wendt et al., 2011). Protein turnover may counteract the resulting short-term E-cad silencing, whereby the balance between recycling and degradation of endocytosed molecules quickly modulates E-cad surface levels (Bryant and Stow, 2004; Huang et al., 2011; Bulgakova et al., 2013; Erami et al., 2015; Bulgakova and Brown, 2016). However, this might be insufficient to protect tissue integrity from a transient but excessive drop in the E-cad levels. Therefore, the robust E-cad expression requires mechanisms for transcriptional activation.

The traditional view is that E-cad expression is activated by constitutive factors in mammalian cells, which are overcome by repressors (Peinado et al., 2004b). This view was contested upon discovery of the binding of the TF FOXA/HNF3 (Forkhead in *Drosophila*) to the E-cad promoter, increasing its expression and driving re-epithelization of breast cancer cells (Liu et al., 2005). This activation system interacts with other E-cad regulatory factors and epigenetic machinery. FOXA suppresses the expression of SLUG, combining E-cad activation with the release of repression (Anzai et al., 2017). FOXA also interacts with p300, which promotes E-cad expression with the two p300-binding sites in the human E-cad promoter (Liu et al., 2005). This interaction suggests the potential role of histone modifications in E-cad transcriptional activation due to the intrinsic histone acetyltransferase (HAT) activity of p300 (Ogryzko et al., 1996; Chan and Thangue, 2001). While p300 can acetylate all histones, it preferentially modifies H3 and H4 and competes for binding with HDAC1 (Ogryzko et al., 1996; Li et al., 2014), making p300 an excellent candidate to counteract the histone deacetylation at the E-cad promoter by HDAC1/2. Finally, FOXA drives E-cad downregulation by interacting with Sp1 and RUNX1/AML1 (Liu et al., 2005; D’Costa et al., 2012). Of particular interest for the data presented below is that in mammalian cells RUNX1/AML1 forms a complex with SUV39H1 [Su(var)3–9 in *Drosophila*], whereas methylation of the H3K9 by SUV39H1 creates a binding site for the heterochromatin protein 1 (HP1) (Lachner et al., 2001; Chakraborty et al., 2003).

Retinoblastoma (Rb) and c-Myc also specifically activate the expression of E-cad promoter through an interaction with the TF AP-2, thus maintaining the epithelial phenotype in mammalian cells (Batsché et al., 1998). Rb interacts with the TF RUNX2, which acts redundantly with closely related RUNX1 in chondrocyte differentiation (Kimura et al., 2010; Gündüz et al., 2012; Komori, 2015). This, alongside RUNX2 and RUNX1 cooperation in tumour cells and RUNX1 recruitment to the E-cad promoter, raises a possibility of similar recruitment of RUNX2, and therefore Rb, to the E-cad promoter (Fowler et al., 2006). Concurrently, Rb depletion induces expression of SLUG and ZEB1, suggesting an indirect effect on E-cad expression (Arima et al., 2008). Additionally to these mechanisms, Rb is likely to modulate epigenetic mechanisms controlling E-cad expression, as it targets the HP1 to gene promoters (Nielsen et al., 2001). This complexity of E-cad transcriptional regulation is further amplified by the involvement of post-transcriptional mechanisms. For example, c-Myc activates E-cad expression, but its substantial overexpression in breast cancer cells leads to post-transcriptional repression of E-cad (Batsché et al., 1998; Cowling and Cole, 2007). While roles of Rb, c-Myc, and Forkhead in the expression of *Drosophila* E-cad are not known, another TF – Grhl2 (Grainyhead in *Drosophila*) – activates E-cad expression in both mammalian and invertebrate cells. In the former, intron-bound Grhl2 promotes E-cad transcription through chromatin looping, playing an essential role in determining an epithelial phenotype (Werth et al., 2010; Xiang et al., 2012). Similarly in flies, Grainyhead has four binding sites in the *shotgun* locus and its overexpression promotes E-cad transcription, as well as that of multiple other genes involved in epithelial maturation (Yao et al., 2017).

The above mechanisms overlook the potential of the cell adhesion molecules (CAMs) in triggering changes in gene expression (Robinson and Moberg, 2011; Dasgupta and McCollum, 2019; Hannezo and Heisenberg, 2019). For example, β-catenin is a TF activated by canonical Wnt signalling (Shang et al., 2017) and induces expression of SLUG in human carcinoma cells (Conacci-Sorrell et al., 2003), meaning that the release of β-catenin from junctions due to E-cad downregulation can reinforce this downregulation. Via α-catenin, E-cad regulates the Hippo pathway by sequestering the YAP protein outside the nucleus, thus explaining the antiproliferative properties of E-cad (Kim et al., 2011; Hannezo and Heisenberg, 2019; Sarpal et al., 2019). α-catenin counteracts β-catenin by inhibiting Wnt-activated transcription (Daugherty et al., 2014). Similarly, p120-catenin antagonises β-catenin-mediated transcriptional activation by interacting with the repressive TF Kaiso (Daniel and Reynolds, 1999; Kourtidis et al., 2013; Schackmann et al., 2013). Curiously, β-catenin expression is inhibited by the Kaiso–p120-catenin complex (Liu et al., 2014), highlighting the reciprocal interactions between transcriptional mechanisms and adhesion molecules. In *Drosophila*, although Kaiso is not present, p120-catenin is nevertheless involved in transcriptional regulation (Stefanatos et al., 2013). As catenins are recruited by E-cad, their availability for either performing nuclear functions or sequestering other TFs outside the nucleus is dependent on E-cad levels. Indeed, E-cad levels impact on transcription of multiple genes involved in a wide range of processes (Soncin et al., 2011). Furthermore, β-catenin recruits the p300 protein, which promotes E-cad expression (Mosimann et al., 2009). This raises the questions of whether E-cad contributes to own gene expression.

## A Feedback Loop Regulating E-Cad Expression Through Par-3, HP1, and STAT92E

The polarity determinant Par-3 (Bazooka in *Drosophila*) is an evolutionarily conserved protein recruited to E-cad adhesion in both mammals and *Drosophila* (Harris and Peifer, 2005; Wei et al., 2005; Iden et al., 2006; Wang et al., 2009; Bulgakova et al., 2013; Xue et al., 2013). Par-3 has six protein–protein interaction domains, making it a versatile scaffold for protein recruitment (McKinley et al., 2012). In *Drosophila*, one of the proteins recruited by Par-3 is the only fly member of the signal transducer and activator of transcription (STAT) protein family, STAT92E (Hou et al., 1996; Yan et al., 1996). This recruitment of STAT92E is necessary for the efficient Janus kinase (JAK)/STAT signalling in *Drosophila* epithelial cells (Sotillos et al., 2008). In human and mouse cells, the loss of Par-3 activates STAT3 signalling, although it is not known if the loss of STAT3 recruitment to E-cad adhesion sites by Par-3 contributes to this activation (McCaffrey et al., 2012; Guyer and Macara, 2015). However, this is very likely, as the loss of E-cad adhesion also leads to STAT3 activation (del Valle et al., 2013).

The canonical signalling pathway comprises STAT translocation into the nucleus after its phosphorylation by the ligand-activated JAK. However, there is growing evidence of non-canonical signalling in both *Drosophila* and mammals, whereby non-phosphorylated STAT stabilises heterochromatin by binding HP1 (Li, 2008; Hixson et al., 2019). HP1 is an evolutionarily conserved non-histone chromatin protein whose best-characterised role is the formation and propagation of heterochromatin (Lomberk et al., 2006; Dialynas et al., 2008; Xu et al., 2014). HP1 is recruited to H3K9me2/3 through SUV39H1, and then itself recruits more SUV39H1 molecules, propagating H3K9 methylation (Lomberk et al., 2006). This is accompanied by DNA methylation in mammalian cells, as both HP1 and SUV39H1 bind DNMTs (Fuks et al., 2003). HP1 was also linked to other functions including transcriptional regulation, where HP1 usually represses transcription by facilitating methylation of H3K9. However, HP1 also localises at specific loci within euchromatin (Dialynas et al., 2008), and there is growing evidence of its association with transcriptionally active regions from *C. elegans* to *Drosophila* and humans (Lu et al., 2000; Piacentini et al., 2003; Mateescu et al., 2008; McMurchy et al., 2017). In *Drosophila*, the active euchromatic regions with HP1 recruitment include developmentally-regulated and heat-shock genes, whose activity correlates with HP1 dosage (Piacentini et al., 2003). Non-phosphorylated STAT92E and HP1 co-localise at euchromatic regions of *Drosophila* polytene chromosomes (Shi et al., 2008). HP1 disperses from chromosomes following hyperactivation of the canonical signalling, suggesting an intricate cross-talk between the two modes of signalling, likely through the availability of non-phosphorylated STAT92E (Xu et al., 2014).

**FIGURE 2:**
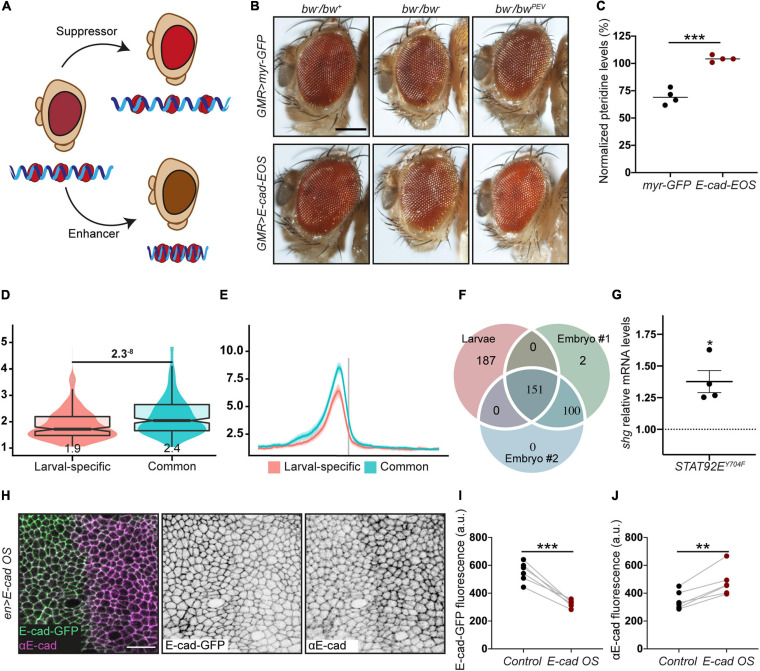
The crosstalk between E-cadherin, STAT92E and HP1. **(A)** A diagram of position effect variegation (PEV) in relation to chromatin compaction. **(B,C)** E-cad overexpression suppresses PEV: examples **(B)** and quantification **(C)**. Scale bar – 0.2 mm, ***p = 0.0001 (unpaired *t*-test). **(D)** Overlapping box-and-violin plots showing the quantification of rBEADS normalised HP1 signal on larval-specific (red) and common (blue) promoters. The *p*-value above – the significance calculated using *U*-test. The value below the boxplots – the mean of the signal. **(E)** The distribution of the rBEADS normalised HP1 signal over transcription start sites (TSS) loci of larval-specific (red) and common (blue) genes. The vertical grey line represents the location of TSS, the plots span 1 kb upstream and downstream from annotated TSS. **(F)** A Venn diagram showing the overlaps between peak loci in the larval dataset and two independent embryonic (16–24 h) replicates. All three peak sets were filtered from peaks overlapping centromeric heterochromatin clusters and non-mappable, repetitive regions, defined based on GEM mappability analyses. modENCODE peak calls have been lifted from dm3 to dm6 genome assembly to match the analyses of larval HP1 peaks. The IDR method with 0.05 *p*-vale threshold was used to collapse the replicates to a common, high confidence peak set. **(G)** *shotgun* expression following overexpression of non-phosphorylatable STAT92E^Y704F^ measured using RT-qPCR and normalised to the expression of house-keeping gene *RpL32*. Four biological replicates are shown as distinct dots with three technical replicates each. *p = 0.02 (one-sample *t*-test comparing to 1). (**H–J)** E-cad-GFP expressed from the endogenous promoter is downregulated following E-cad overexpression (E-cad OS) from a heterologous UAS promoter. A representative image **(H)** shows endogenously expressed E-cad-GFP visualised with native GFP fluorescence (green, left; grey, middle) and total E-cad visualised with antibody staining (magenta, left; grey, right), scale bar – 10 μm. Levels of endogenously expressed E-cad-GFP are quantified in panel **(I)** and total E-cad levels in panel **(J)**. **p = 0.0018 and ***p = 0.0003 (paired *t*-test).

As STAT92E promotes heterochromatin gene silencing in a dose-dependent manner (Shi et al., 2008), we hypothesised that an increase of E-cad levels would lead to STAT92E sequestration at adhesion sites, making it unavailable to bind HP1 and promote heterochromatin formation alongside other functions (Figure 1C). We examined the effects of E-cad overexpression on heterochromatic gene silencing monitored using position-effect variegation (PEV). In this assay, a gene is translocated into a peri-heterochromatic region where its expression is silenced in a stochastic pattern (Figure 2A). This silencing depends on the spreading of heterochromatin: if a protein normally promotes heterochromatin formation, its loss would reduce the amount of heterochromatin and suppress the variegation (=loss of silencing of the translocated gene, Figure 2A; Elgin and Reuter, 2013). We overexpressed E-cad in the *Drosophila* eye using the UAS–Gal4 system with *GMR*::Gal4, which expresses Gal4 in all retinal cells (Hay et al., 1997). We used the variegating allele of the *brown* gene (*bw*^PEV^), necessary to produce the red pigment pteridine. Overexpression of E-cad increased the normalised eye pigmentation with pteridine to 104% from 69% in the control (Figures 2B,C and Supplementary Figure 1). Therefore, E-cad suppresses PEV in the eye, supporting that elevated E-cad inhibits heterochromatin formation.

Next, we sought to determine what genes might be regulated through our hypothetical mechanism and examined the HP1 localisation using publicly available ChIP-seq datasets generated using third instar larvae (Riddle et al., 2011), which include epithelial monolayers of imaginal discs. There were distinct peaks of HP1 overlapping with promoters of 456 genes outside of heterochromatin clusters and non-mappable (non-unique, repeatable) regions (Figures 2D,E and Supplementary Figure 2). We compared the larval HP1 peaks with two independent replicates from an earlier developmental stage – embryos at 16–24 h of development (modENCODE Consortium, Roy et al., 2010). Roughly half of the HP1 peaks in the larval dataset (155 peaks mapping to 151 loci) were common to all three datasets, while another half (187 peaks/loci) were larval-specific (Figure 2F). The common peaks (potentially, developmentally conserved) mostly mapped to bi-directional promoters, so that 155 peaks mapped to a total of 280 genes. These genes are enriched for housekeeping terms: regulation of primary and macromolecule metabolic processes (Supplementary Figure 3, terms 7–11); and mitotic cell cycle and cell cycle regulation (Supplementary Figure 3, terms 2–6). In contrast, the larval-specific peaks, which map to promoters of 178 genes, are enriched for terms related to epithelial tissue: morphogenesis of epithelium, actin filament organisation, epithelium development and imaginal disc development (Supplementary Figure 3, terms 19–24). The *shotgun* promoter overlapped a larval-specific HP1 peak with a signal value of 9.83 (207th promoter rank). This peak is consistent with two potential mechanisms for HP1 recruitment – via Su(var)3–9 or one of the two fly Rb-family proteins. Therefore, the modulation of HP1 function downstream of E-cad and non-canonical STAT signalling might alter the E-cad expression.

Indeed, overexpression of the non-phosphorylatable STAT92E (STAT92E^Y704F^), which acts only in the non-canonical signalling (Karsten et al., 2006; Xu et al., 2014), increased expression of the *shotgun* gene (Figure 2G). Such an increase is consistent with the model whereby an unprogrammed drop in E-cad levels releases STAT92E, which leads to a consequent increase of E-cad expression and thus restores E-cad levels (Figure 1C). We observed that overexpression of E-cad from a heterologous UAS promoter leads to downregulation of the protein expressed from the endogenous locus (Figures 2H–J). This might be accompanied by simultaneous upregulation of the canonical JAK/STAT signalling as observed for STAT3 activation in some human cancers (Loh et al., 2019). We postulate that other genes with larval-specific HP1 peaks behave similarly to E-cad. Then, the activation of non-canonical STAT signalling following a stochastic drop in E-cad levels would reinforce the epithelial nature of the cells as many of these genes are involved in epithelial morphogenesis.

## Outlook/Perspective

E-cad levels at the cell surface play an important role in determining cellular properties: cell shape, response to signalling, cell division, and neighbour exchange to name just a few (Lecuit and Lenne, 2007; Rübsam et al., 2017; Mendonsa et al., 2018). Not surprisingly, E-cad expression is subjected to complex regulation by multiple interconnected mechanisms. However, to respond to the cell’s needs and modulate E-cad levels accordingly, information about the E-cad levels at the surface must be transferred to the nucleus. Here, we propose one such mechanism, whereby surface E-cad feeds back to the nucleus through competition for STAT92E binding. We hypothesise that this feedback stabilises E-cad transcription enabling robust and resilient cell–cell adhesion. Importantly, this provides the cell with a tumour-suppressive mechanism, as heterochromatin formation, which is likely to follow a drop in E-cad levels, is linked with cellular senescence (Narita et al., 2003). This contrasts another feedback mechanism whereby the release of β-catenin from adhesion sites leads to SLUG expression and reinforces E-cad silencing in human cells. Therefore, depending on the context E-cad expression may be stabilised or silenced through feedback interactions. It is tempting to speculate that further mechanisms of information transfer from the cell surface to the nucleus exist – not unlike the multiple and intertwined mechanisms for regulation of E-cad transcription. A comprehensive understanding of E-cad transcriptional regulation requires the discovery and in-depth analysis of such mechanisms.

## Data Availability Statement

The datasets presented in this study can be found in online repositories. The names of the repository/repositories and accession number(s) can be found below: http://data.modencode.org/, 3187; http://data.modencode.org/, 3188; http://data.modencode.org/, 3391; http://data.modencode.org/, 3392.

## Author Contributions

NAB and MRM performed the experiments and wrote the manuscript. NAB and PAS did the bioinformatics analyses. All authors contributed to the article and approved the submitted version.

## Conflict of Interest

PAS was a shareholder and the managing director of the company SmartImmune Ltd. The remaining authors declare that the research was conducted in the absence of any commercial or financial relationships that could be construed as a potential conflict of interest.
