# Supplementary material for: Interactions and Feedbacks in E-Cadherin Transcriptional Regulation
Source: Front Cell Dev Biol. 2021 Jun 28;9:701175. doi: 10.3389/fcell.2021.701175 (PMC8273600; doi:10.3389/fcell.2021.701175)
Supplement: Supplementary file 1 [file Data_Sheet_1.pdf]

Supplementary Information

Supplementary materials and methods

Fly stocks

The following fly stocks were used in this study (with Bloomington Drosophila Stock Center numbers when available): *GMR*::Gal4 (1104), bw^1 (245), bw^{VDe2} (bw^{PEV}, 1582), *UAS*::myr-GFP (32200), *Act5C*::Gal4 (24414), *tubulin*::Gal80^{ts} (7019), *en*::Gal4 (30564), *shg*::E-cad-GFP (60584), *UAS*::E-cad (65589), *UAS*::E-cad-EOS (Cavey et al., 2008), and *UAS*::STAT92E^{Y704F}-GFP (Karsten et al., 2006). All crosses were kept on standard media at 25°C.

Position effect variegation

As all transgenic constructs carried the *mini-white* gene and we could not use the variegated alleles of the *white* gene, we employed the variegating allele of the *brown* gene (bw^{PEV}), necessary for the production of the red pigment pteridine (drosopterin). The protocol to measure drosopterin levels was adapted from (Hainey, 1979). As the red and brown patches in mosaic eyes cannot be distinguished and scored visually, the absorbance of the pigment solution at 470 nm (OD_{470}) was measured with a spectrophotometer (Beckman) as it scales linearly with pigment levels (Supplementary Figure 1) (Hainey, 1979). The flies were beheaded on a CO_2 pad with a razor blade, and heads were cut longitudinally in halves. Individual heads were incubated with 30% ethanol in deionized water, pH 2 for 24 hours at room temperature. Three heads were measured independently per genotype, with the clear solvent used as a blank. As E-cad overexpression mildly increases the overall eye size, we normalized the measured amount of red pigment to that produced in the absence of variegation in bw^+/bw^- flies. Namely, the measurements for eyes overexpressing E-cad or myr-GFP (control) in bw^+ and bw^{PEV} background were normalized to corresponding measurements on bw^- background. Then, measurements on bw^{PEV} background were expressed as a percentage of those on bw^+. The values were compared using the unpaired two-tailed t-test following the normality Shapiro-Wilk test.

RT-qPCR

STAT92E^{Y704F} and myr-GFP were expressed using *Act5C*::Gal4 for 24 hours following temperature shift to 25°C to inactivate *tubulin*::Gal80^{ts}. mRNA was isolated from third instar larvae wing discs using the NucleoSpin RNA XS kit (Machery-Nagel) and cDNA was generated using the High Capacity RNA-to-cDNA Kit (Applied Biosystems). The following

primer pairs were used: GACGCTTCAAGGGACAGTATCTG and AAACGCGGTTCTGCATGAG for *RpL32* (Bina et al., 2010), and TCCATGGTCGGAAAATGCCCA and AGTACTGAAAGTCGCGCTCC for *shg*. Ct values were obtained from SYBR fluorescence using thresholds determined from the standard curves (Larionov et al., 2005). Reactions were carried out in 3 technical replicates per each biological replicate (15 wing discs). Expression levels were determined by the $2^{-\Delta\Delta CT}$ method (Schmittgen & Livak, 2008). For each biological replicate, Ct values were averaged across technical replicates and the average Ct value of *RpL32* was subtracted. This result (ΔCT) was normalized by subtracting the average ΔCT of the control genotype (myr-GFP), producing ΔΔCT, which was converted as $2^{-\Delta\Delta CT}$. The resulting values were compared using the one-sample t-test following the normality Shapiro-Wilk test.

Immunostaining and imaging of wing discs

UAS::E-cad was expressed using *engrailed*::Gal4 in animals with GFP-tagged E-cad expressed from the endogenous locus (*shg*::E-cad-GFP). Third instar larvae were dissected and cuticles with attached imaginal discs were fixed for 15 minutes with 4% formaldehyde (Sigma, F8775) in PBS (Phosphate Buffer Saline, Sigma-Aldrich, P4417) at room temperature, and then washed with PBS with 0.1% Triton X-100 (Thermo Fisher, A16046, PBST). Cuticles were incubated with primary rat anti-E-cad antibody (DCAD2, DSHB, 1:200) and 1% Bovine Serum Albumin (BSA, New England Biolabs, B9000S) in PBST overnight at 4°C, washed in PBST, incubated for 2 hours at room temperature with the secondary anti-rat-Cy3 antibody (Jackson ImmunoResearch, 712-165-153) and 1% BSA in PBST, and washed in PBST again. Finally, discs were separated from the cuticles and mounted in Vectashield (Vector Labs, H-1000).

Microscopy was done using an upright Olympus FV1000 confocal microscope with the 60x/1.40 NA objective. A z-stack of 6 slices spaced by 0.38 μm was acquired capturing the complete span of the Adherens Junctions. The images were at 16-bit depth in Olympus binary image format. For the analysis z-stacks with signal of GFP fluorescence were projected using the "average intensity" algorithm in Fiji (https://fiji.sc). These projections were used to generate masks using the Tissue Analyzer plugin in Fiji (Aigouy et al., 2010).

Areas overexpressing E-cad (*engrailed*-positive) and non-overexpressing (*engrailed*-negative) were manually cropped and analysed individually. The binary masks were

generated from each area and used to measure the fluorescence intensity of average projections using our in-house Matlab script (https://github.com/nbul/Intensity). Individual objects (cells) and their boundaries were identified from each binary mask. Then individual junctions between tricellular vertices were dilated using a diamond-shaped morphological structural element of size 3 to encompass the XY spread of the E-cad signal. All the values were averaged to produce single values per area per wing disc thus testing biological replicates. The resulting values were compared using the paired t-test following the normality Shapiro-Wilk test.

ChIP-seq analyses

The following datasets were used (modEncode IDs): 3187, 3188, 3391, 3392 (all from embryos, 16-24 hours after egg laying), 4936 (3d instar larvae).

Alignment to the reference genome

Reads from ChIP-seq libraries were aligned to the *Drosophila melanogaster* reference genome (BDGP6.28/dm6) using BWA v. 0.7.17 (Li & Durbin, 2010) with default settings (BWA-backtrack algorithm). The SAMtools v. 1.3.1 'view' utility was used to convert the alignments to BAM format.

Peak calls

ChIP-seq peaks were called for each of ChIP replicate using MACS v.2.2.7.1 (Feng et al., 2011) with permissive 0.05 FDR (q-value) cut-off and insert size of 150 bp against the corresponding ChIP-seq inputs. IDR (Irreproducible Discovery Rate) v.2.0.4.2 with a hard IDR threshold of 0.05 was used to collapse replicates into a single, high confidence peak call. To make sure these peaks are not ChIP artefacts, we tested for their overlap with high occupancy of transcription factors (HOT) regions (Chen et al., 2014; Wreczycka et al., 2019). No significant overlap suggested that peaks are not artefacts caused by particularly open chromatin on "hyperchipable" regions.

Filtering peak calls and overlapping promoter analyses

To analyse only HP1 peaks with a potential to specifically regulate genes, we excluded the following peaks from the further analyses: 1) located in centromeric clusters; and 2) located in repetitive, non-mappable regions. We arbitrarily defined centromeric clusters as following:

2L:22160000-23513712, 2R:1-5750000, 3L:23000000-28110227, 3R:1-4156000, 4:1-134813. To find low-mappability regions we produced a mappability track from the *D. melanogaster* reference genome (BDGP6.28) using the GEM mappability method (Derrien et al., 2012).Then we tiled mappability into 100bp regions and discarded peaks overlapping with the loci of <0.5 mappability index.

Peak calls overlapping coding gene promoters

Coding gene annotation (DGP6.28.100) from Ensembl was used to define promoter loci. We defined promoters as region 1000bp upstream to 200bp downstream from annotated coding gene transcription start site (TSS). Promoters directly overlapping HP1 peaks were ranked by ChIP signal value and further used for analyses and visualizations. For gene ontology overrepresentation, testing and visualization we used g:GOSt utility from g:Profiler suite (https://biit.cs.ut.ee/gprofiler/gost).

Venn diagrams for peaks overlaps

Two independent replicated and IDR collapsed HP1 embryo peak calls were downloaded from modENCODE repository (https://personal.broadinstitute.org/anshul/projects/fly/peaks_macs/idrOptimalBlacklistFiltered/). Because of reference genome version mismatch, these peaks were lifted over from dm3 to BDGP6.28/dm6 genome version using the UCSC Batch Coordinate Conversion (liftOver) utility and chain file (http://hgdownload.soe.ucsc.edu/goldenPath/dm3/liftOver/dm3ToDm6.over.chain.gz). To produce the overlap plots, we found the loci occupied by any of the three peak calls, and then categorize them as overlapping with three or two peak calls, or unique to a single peak call. We visualised these numbers on Venn diagrams.

ChIP-seq normalisation and summarised visualization

We used aligned reads, with BWA mapping quality >=10 to generate the pileup and normalised tracks. The normalized ChIP-seq coverage tracks were generated using the rBEADS implementation of BEADS algorithm (Cheung et al., 2011; Przemyslaw Stempor, 2014). For rBEADS controls, we used a combined ChIP input for the HP1 replicates and GEM mappability track. BEADS normalised tracks were used for visualization using IGV and summarised region visualization with SeqPlots (Stempor & Ahringer, 2016).

Supplementary Figures

Supplementary Figure 1: OD_{470} scales linearly with levels of pteridine.

A – Measures of OD_{470} of pteridine extracts (Y-axis) from one or two heads (X-axis) of the genotypes indicated in the legend. Both white (w^-) and brown (bw^-) mutants lack pteridine. The line corresponds to linear regression using wild type ($w^+; bw^+$). N = two independent biological replicates per genotype and number of heads.

Figure S1

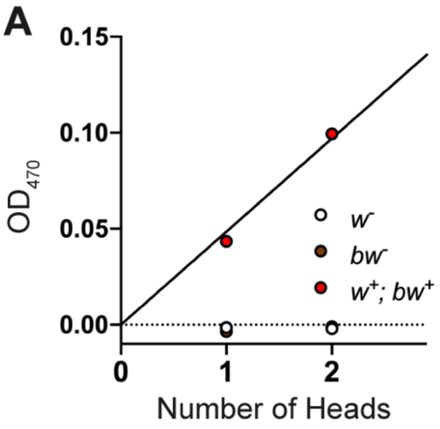

Supplementary Figure 2. HP1 localizes to a set of coding genes promoters.

A – Overlapping box-and-violin plots showing the quantification of rBEADS normalised HP1 signal on larva specific (shown in red) and common (shown in blue) promoters. The p-value above represents the statistical significance calculated using U-test. The value below the boxplots represents the mean of the signal.

B – The distribution of the rBEADS normalised HP1 signal over transcription start sites (TSS) loci of larval-specific (red) and common (blue) genes. The vertical grey line represents the location of TSS, the plots span 1kb upstream and downstream from annotated TSS.

C – Heatmaps showing the same loci as B. Each row of the heatmap represents a single promoter locus. The top of the heatmaps shows larval-specific and bottom – common TSS regions. The sets are ordered based on the rank (signal value) of the overlapping HP1 peaks. Replicates are shown separately on the left and right panels.

Figure S2

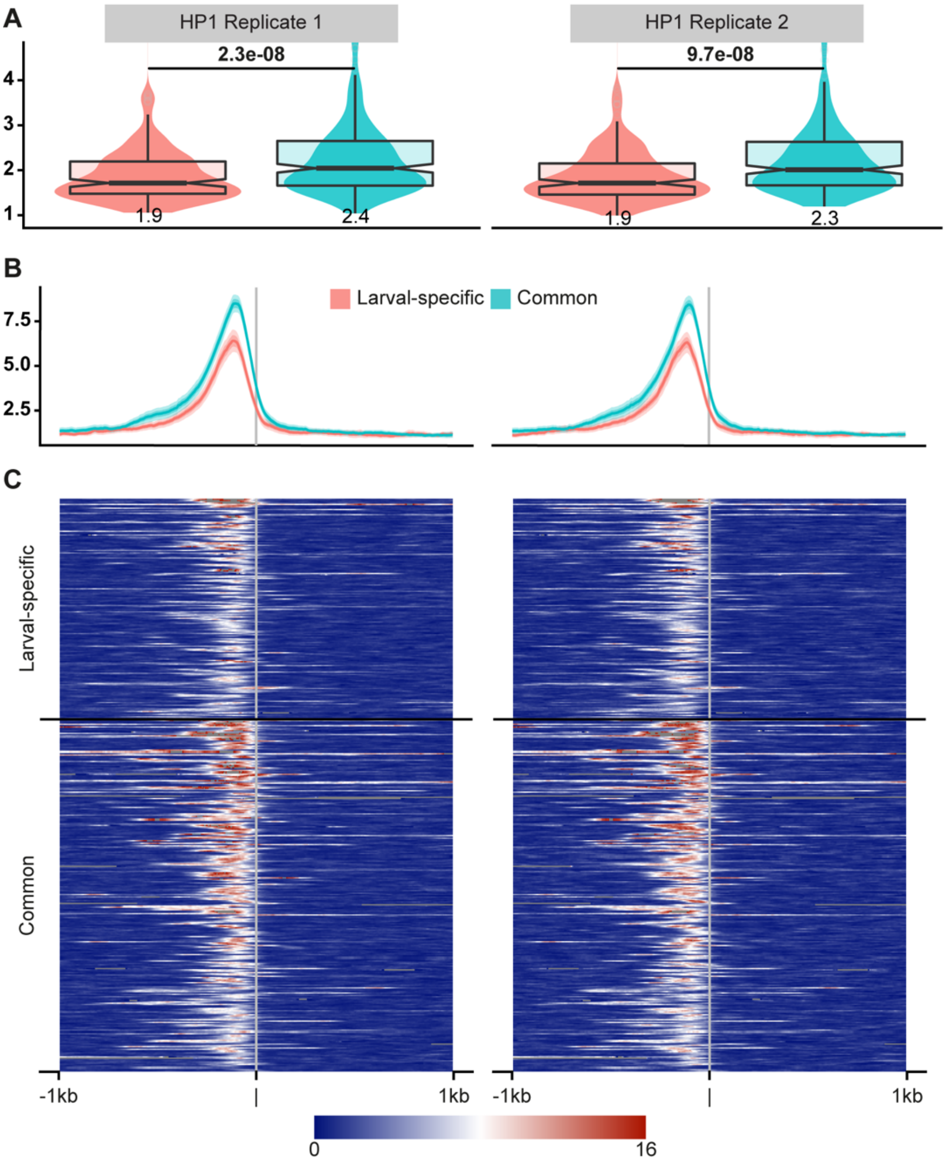

Supplementary Figure 3. The visualization and a comparative table of GO terms.

A – Manhattan plots of GO terms, where terms close in hierarchical structure are located together on the X-axis, while the Y-axis represents the negative log scaled p-values (a higher value denotes a bigger significance). The top plot shows common genes (an HP1 promoter peak in both embryonic and larval experiments), while the bottom plot shows larval-specific genes. **B** – Terms and their significance in the two sets depicted in A. The numbered rows in the table correspond to numeric annotations on Manhattan plots (an interactive version at https://biit.cs.ut.ee/gplink/l/rLmb8A3MRY).

Figure S3

A

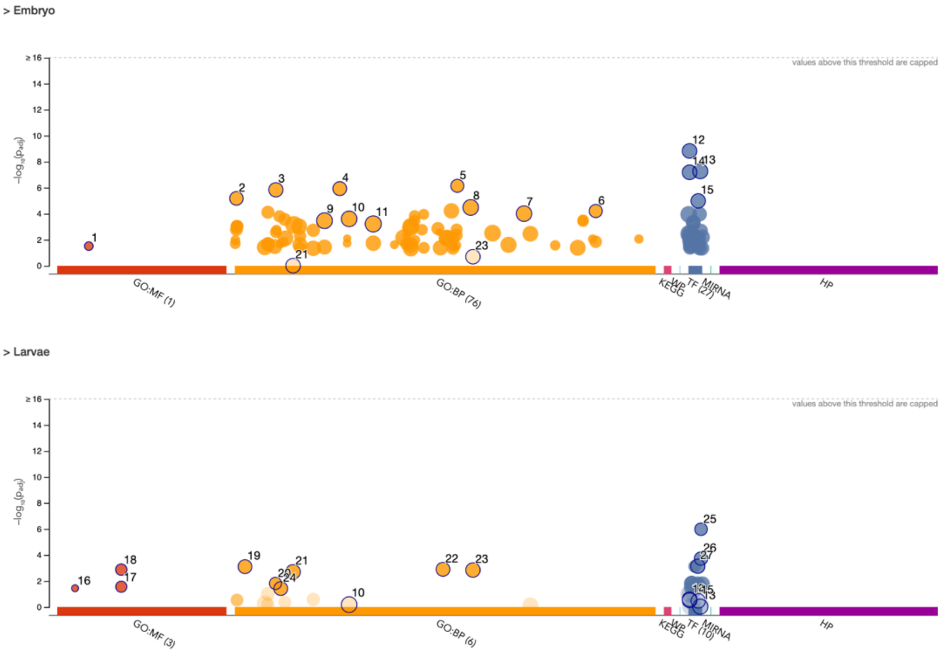

B

ID	Source	Term ID	Term Name	p_{adj} (Embryo)	p_{adj} (Larvae)
1	GO:MF	GO:0008574	ATP-dependent microtubule motor activity, plu...	3.086×10^{-2}	
2	GO:BP	GO:0000278	mitotic cell cycle	6.670×10^{-6}	~~1.000~~
3	GO:BP	GO:0007049	cell cycle	1.447×10^{-6}	~~1.000~~
4	GO:BP	GO:0022402	cell cycle process	1.175×10^{-6}	~~1.000~~
5	GO:BP	GO:0051726	regulation of cell cycle	7.188×10^{-7}	~~1.000~~
6	GO:BP	GO:1903047	mitotic cell cycle process	6.205×10^{-5}	~~1.000~~
7	GO:BP	GO:0080090	regulation of primary metabolic process	9.898×10^{-5}	~~1.000~~
8	GO:BP	GO:0060255	regulation of macromolecule metabolic process	3.292×10^{-5}	~~1.000~~
9	GO:BP	GO:0019222	regulation of metabolic process	3.449×10^{-4}	~~1.000~~
10	GO:BP	GO:0031323	regulation of cellular metabolic process	2.492×10^{-4}	~~6.461×10^{-1}~~
11	GO:BP	GO:0034641	cellular nitrogen compound metabolic process	6.069×10^{-4}	~~1.000~~
12	TF	TF:M07142	Factor: BEAF-32A; motif: NNNMNAMTATCGATA	1.500×10^{-9}	~~2.480×10^{-1}~~
13	TF	TF:M12368	Factor: SRYDELTA; motif: YTATCGATAKTT	5.427×10^{-8}	~~9.368×10^{-1}~~
14	TF	TF:M12357	Factor: BEAF32; motif: NTATCGATAKTTNN	6.570×10^{-8}	~~3.028×10^{-1}~~
15	TF	TF:M07149	Factor: pnr; motif: TATCGATAGNY	1.034×10^{-5}	~~3.341×10^{-1}~~
16	GO:MF	GO:0004708	MAP kinase kinase activity		3.569×10^{-2}
17	GO:MF	GO:0019901	protein kinase binding	~~1.000~~	2.729×10^{-2}
18	GO:MF	GO:0019900	kinase binding	~~1.000~~	1.323×10^{-3}
19	GO:BP	GO:0002009	morphogenesis of an epithelium	~~1.000~~	7.864×10^{-4}
20	GO:BP	GO:0007015	actin filament organization	~~1.000~~	1.510×10^{-2}
21	GO:BP	GO:0009888	tissue development	~~9.841×10^{-1}~~	1.967×10^{-3}
22	GO:BP	GO:0048729	tissue morphogenesis	~~1.000~~	1.249×10^{-3}
23	GO:BP	GO:0060429	epithelium development	~~2.013×10^{-1}~~	1.421×10^{-3}
24	GO:BP	GO:0007444	imaginal disc development	~~1.000~~	3.791×10^{-2}
25	TF	TF:M07665_1	Factor: GAGA; motif: MCTCTCTCCC; match cla...	~~1.000~~	1.038×10^{-6}
26	TF	TF:M02323_1	Factor: Trl; motif: YYNCTCTCTN; match class: 1	~~1.000~~	1.869×10^{-4}
27	TF	TF:M07901	Factor: org-1; motif: AGGTGTGAN	~~1.000~~	7.310×10^{-4}

Supplementary References

Aigouy, B., Farhadifar, R., Staple, D. B., Sagner, A., Roper, J. C., Julicher, F., & Eaton, S. (2010). Cell flow reorients the axis of planar polarity in the wing epithelium of Drosophila. *Cell*, *142*(5), 773–786. https://doi.org/10.1016/j.cell.2010.07.042

Bina, S., Wright, V. M., Fisher, K. H., Milo, M., & Zeidler, M. P. (2010). Transcriptional targets of Drosophila JAK/STAT pathway signalling as effectors of haematopoietic tumour formation. *EMBO Reports*, *11*(3), 201–207. https://doi.org/10.1038/embor.2010.1

Chen, R. A.-J., Stempor, P., Down, T. A., Zeiser, E., Feuer, S. K., & Ahringer, J. (2014). Extreme HOT regions are CpG-dense promoters in C. elegans and humans. *Genome Research*, *24*(7), 1138–1146. https://doi.org/10.1101/gr.161992.113

Cheung, M.-S., Down, T. A., Latorre, I., & Ahringer, J. (2011). Systematic bias in high-throughput sequencing data and its correction by BEADS. *Nucleic Acids Research*, *39*(15), e103. https://doi.org/10.1093/nar/gkr425

Derrien, T., Estellé, J., Sola, S. M., Knowles, D. G., Raineri, E., Guigó, R., & Ribeca, P. (2012). Fast Computation and Applications of Genome Mappability. *PLOS ONE*, *7*(1), e30377. https://doi.org/10.1371/journal.pone.0030377

Hainey, S. (1979). *Variation in the eye pigment pathways of Drosophila melanogaster*. https://era.ed.ac.uk/handle/1842/14956

Karsten, P., Plischke, I., Perrimon, N., & Zeidler, M. P. (2006). Mutational analysis reveals separable DNA binding and trans-activation of Drosophila STAT92E. *Cell Signal*, *18*(6), 819–829. https://doi.org/10.1016/j.cellsig.2005.07.006

Larionov, A., Krause, A., & Miller, W. (2005). A standard curve based method for relative real time PCR data processing. *BMC Bioinformatics*, *6*, 62. https://doi.org/10.1186/1471-2105-6-62

Li, H., & Durbin, R. (2010). Fast and accurate long-read alignment with Burrows-Wheeler transform. *Bioinformatics (Oxford, England)*, *26*(5), 589–595. https://doi.org/10.1093/bioinformatics/btp698

Przemyslaw Stempor. (2014). *RBEADS - The R implementation of Bias Elimination Algorithm for Deep Sequencing*. Zenodo. https://doi.org/10.5281/zenodo.11427

Schmittgen, T. D., & Livak, K. J. (2008). Analyzing real-time PCR data by the comparative CT method. *Nature Protocols*, *3*(6), 1101–1108. https://doi.org/10.1038/nprot.2008.73

Stempor, P., & Ahringer, J. (2016). SeqPlots—Interactive software for exploratory data analyses, pattern discovery and visualization in genomics. *Wellcome Open Research*, *1*, 14. https://doi.org/10.12688/wellcomeopenres.10004.1

Wreczycka, K., Franke, V., Uyar, B., Wurmus, R., Bulut, S., Tursun, B., & Akalin, A. (2019). HOT or not: Examining the basis of high-occupancy target regions. *Nucleic Acids Research*, *47*(11), 5735–5745. https://doi.org/10.1093/nar/gkz460
